# Phosphatidic acid biosynthesis in the model organism yeast *Saccharomyces cerevisiae* - a survey

**DOI:** 10.1016/j.bbalip.2021.158907

**Published:** 2021-02-18

**Authors:** Karin Athenstaedt

**Affiliations:** Institute of Molecular Biosciences, University of Graz, Humboldtstrasse 50/2, 8010 Graz, Austria

**Keywords:** Acyltransferase, Glycerolipid, Lipid droplet, Phosphatidic acid, *Saccharomyces cerevisiae*, Yeast

## Abstract

Phosphatidic acid biosynthesis represents the initial part of de novo formation of all glycerophospholipids (membrane lipids) as well as triacylglycerols (storage lipids), and is thus the centerpiece of glycerolipid metabolism. The universal route of phosphatidic acid biosynthesis starts from the precursor glycerol-3-phosphate and comprises two consecutive acylation reactions which are catalyzed by a glycerol-3-phosphate acyltransferase and a 1-acyl glycerol-3-phosphate acyltransferase. In addition, yeast and mammals harbor a set of enzymes which can synthesize phosphatidic acid from the precursor dihydroxyacetone phosphate. In the present review our current knowledge about enzymes contributing to phosphatidic acid biosynthesis in the invaluable model organism yeast, *Saccharomyces cerevisiae*, is summarized. A special focus is laid upon the regulation and the localization of these enzymes. Furthermore, research needs for a deeper insight into the high complexity of phosphatidic acid biosynthesis and consequently the entire lipid metabolic network is presented.

## 1 Introduction

**Fig. 1:**
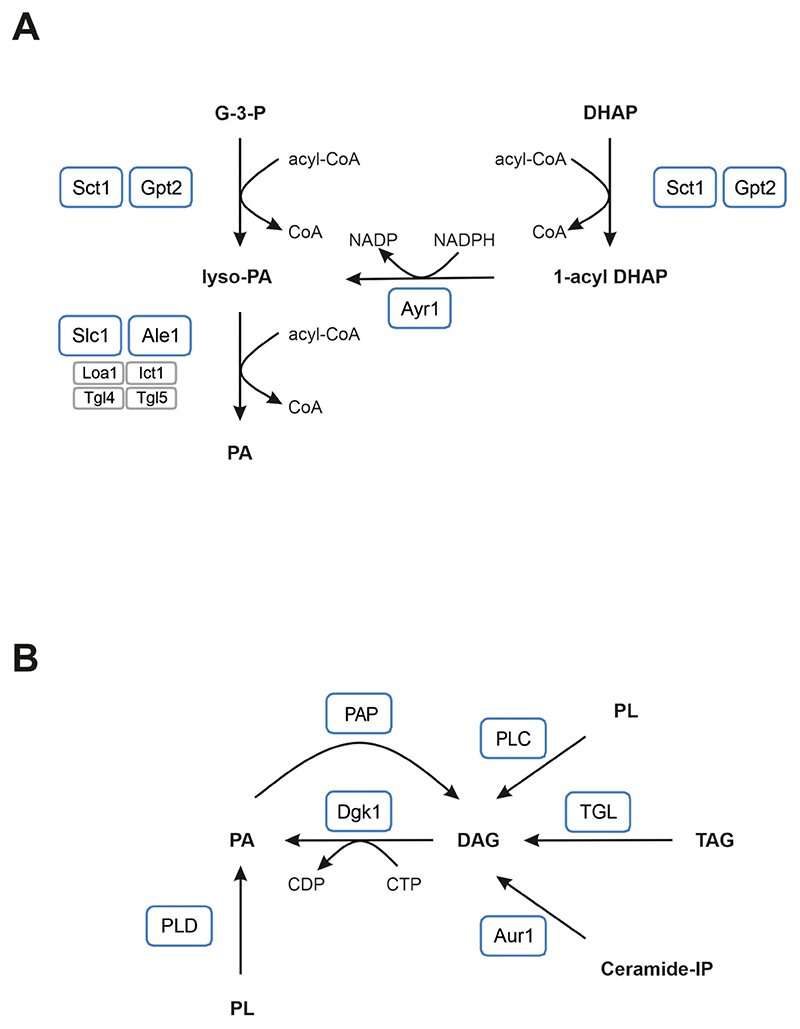
Pathways of phosphatidic acid formation in yeast. (A) De novo phosphatidic acid (PA) is synthesized either via the glycerol-3-phosphate (G-3-P) pathway (left) or the dihydroxyacetone phosphate (DHAP) pathway (right). Major enzymes catalyzing the respective reactions of these pathways are framed in blue, whereas minor enzymes are framed in grey.(B) Alternatively, PA is formed from glycerophospholipids (PL) which are cleaved by a phospholipase D (PLD), or by phosphorylation of diacylglycerol (DAG) by the diacylglycerol kinase Dgk1. DAG is derived by the degradation of PL (mediated by a phospholipase C; PLC), of PA (mediated by a phosphati-date phosphatase; PAP), or of triacylglycerols (TAG) (mediated by a TAG lipase; TGL). Similarly, the turnover of ceramide-inositol phosphate (Ceramide-IP) mediated by Aur1 yields DAG. 1-acyl DHAP, 1-acyl dihydroxy - acetone phosphate; lyso-PA, lyso-phosphatidic acid. For further information see text.

Phosphatidic acid (PA) plays a central role in lipid metabolism, because it is required for de novo synthesis of all glycerophospholipids and triacylglycerols. In addition, PA is involved in cell signaling, membrane fusion and fission events, as well as it functions as a cellular pH sensor [1]. Another interesting aspect which is currently in focus of lipid research is the role of PA in lipid droplet biogenesis. Evidence has been provided that the local concentration of PA in the endoplasmic reticulum (ER) plays a critical role in this process [[2,3]; reviewed in [4,5]]. This aspect is of highest interest from the pathologic point of view, because defects in lipid droplet biogenesis/maintenance are related to severe lipid storage diseases. De novo PA is synthesized from the precursor glycerol-3-phosphate by two consecutive acylation reactions (Fig. 1A). The first and rate determining acylation reaction occurs at the *sn*-1 position and converts glycerol-3-phosphate to 1-acyl glycerol-3-phosphate (lyso-phosphatidic acid; lyso-PA). This reaction is immediately followed by the second acylation reaction at the *sn*-2 position yielding PA. The enzymes catalyzing these consecutive acylation reactions are a glycerol-3-phosphate acyltransferase (GPAT) and a 1-acyl glycerol-3-phosphate acyltransferase (AGPAT), respectively. Interestingly, with few exceptions [6] in eukaryotic organisms at least two enzymes of each type of acyltransferase exist [reviewed in, e.g., [7–10]]. This is similarly true for the budding yeast *Saccharomyces cerevisiae* (in the following termed “yeast”), an invaluable model organism especially in lipid research. In this yeast two GPATs with overlapping function are expressed which are named Sct1 and Gpt2 [11]. Similarly, for the second acylation reaction converting lyso-PA to PA two major AGPATs have been described, Slc1 [12] and Ale1 [13–17]. However, also some other enzymes exhibit minor AGPAT activity, but their contribution to PA biosynthesis seems to be restricted to specific conditions such as, e.g., sporulation or growth in the presence of organic solvents [18–21].

The formation of PA from the precursor glycerol-3-phosphate represents the main route of PA biosynthesis. However, like mammals, yeast can also utilize dihydroxyacetone phosphate as a substrate to synthesize PA (Fig. 1A). In the respective pathway which is known as dihydroxyacetone phosphate pathway, first an acyl-chain is linked to the hydroxyl-group of dihydroxyacetone phosphate yielding 1-acyl dihydroxyacetone phosphate. Next, the keto-group of 1-acyl dihydroxyacetone phosphate is reduced in an NADPH dependent reaction resulting in the formation of lyso-PA. Since lyso-PA is also the intermediate of the glycerol-3-phosphate pathway, by this latter reaction the dihydroxyacetone phosphate pathway joins the glycerol-3-phosphate pathway. Accordingly, a final acylation reaction converts lyso-PA to PA. The set of enzymes mediating the reactions of the dihydroxyacetone phosphate pathway comprises two different types of acyltransferases, i.e., a dihydroxyacetone phosphate acyltransferase and an AGPAT, and an NADPH dependent 1-acyl dihydroxyacetone phosphate reductase. In yeast, both GPATs Sct1 and Gpt2 accept not only glycerol-3-phosphate as a substrate, but are also able to initiate PA formation from the precursor dihydroxyacetone phosphate [11,22]. Similarly, the acyltransferases catalyzing the second acylation reaction are the same for both pathways, since this reaction is common to both pathways. In contrast, the reduction reaction which converts 1-acyl dihydroxyacetone phosphate to lyso-PA is specific for the dihydroxyacetone phosphate pathway and catalyzed by the 1-acyl dihydroxyacetone phosphate reductase Ayr1 [23]. To date, Ayr1 is the only 1-acyl dihydroxyacetone phosphate reductase identified in yeast, but residual reductase activity in an *ayr1*Δ strain indicates that Ayr1 has at least one counterpart which remains to be identified.

The GPATs Sct1 and Gpt2, as well as the AGPAT Slc1 are members of the GPAT/AGPAT protein family. This protein family is characterized by four short conserved amino acid stretches which are known as motifs I, II, III and IV [24]. Among these motifs, motif I shows the strongest conservation and comprises an invariant histidine and aspartic acid which are separated by four amino acids. Due to this characteristic, this motif is also known as HX_4_D motif. During catalysis, the conserved histidine and aspartic acid form a charge-relay system, which increases the nucleophilicity of the hydroxyl-group of glycerol-3-phosphate. In the following, the activated hydroxyl-group attacks the carboxyl-group of acyl-CoA and attaches the acyl-chain to the glycerol backbone. Interestingly, in both yeast GPATs, Sct1 and Gpt2, the conserved histidine and aspartic acid are separated by five amino acids instead of four [6]. The significance of the bigger distance between the conserved amino acids in this motif in the yeast GPATs compared to other acyltransferases is currently not known. Like motif I, also motif IV is involved in catalysis. Motif II and motif III, on the other hand, are required for substrate binding. Mutations in these motifs are equally critical for the catalytic function of the enzyme. For instance, in the yeast GPATs the conserved sequence of motif III comprises the amino acids “FPEGGSHDR”. Replacement of either of the two glycines in this motif by a bulky amino acid inactivates Sct1 and Gpt2 [11,25–27]. Another striking difference between Sct1 and Gpt2, and GPATs from other organisms is the distance between motif II and motif III. Whereas in GPATs of mammals and prokaryotes these two motifs are separated by 20 to 30 amino acids, in Sct1 and Gpt2 this intervening sequence is approximately 120 amino acids in length [6]. This difference may explain why the yeast GPATs accept not only glycerol-3-phosphate as a substrate, but also dihydroxyacetone phosphate.

The second major AGPAT Ale1 is a member of the membrane bound O-acyltransferase (MBOAT) protein family. This group of proteins comprises quite diverse acyltransferases which use not only lipids as a substrate, but also proteins. This diversity is most likely also the reason why the common characteristic of the MBOAT protein family is limited to one invariable histidine. However, whether and how this histidine participates in catalysis is currently not clear.

Cell proliferation strictly relies on de novo synthesis of PA. However, also formation of PA via alternative routes plays an important role in cell metabolism, for instance in cell signaling, during sporulation, and in membrane fission and fusion events. One of these additional routes of PA formation comprises the cleavage of glycerophospholipids by a phospholipase D, an enzyme cleaving off the head-group of a glycer-ophospholipid distal of the phosphate group which results directly in PA (Fig. 1B). Alternatively, PA is formed by phosphorylation of diacylglycerol, which is derived by degradation of glycerophospholipids (mediated by a phospholipase C), PA (mediated by a phosphatidate phosphatase), or triacylglycerols (mediated by a triacylglycerol lipase) (Fig. 1B). Similarly, the turnover of sphingolipids yields diacylglycerol as a side product. The phosphorylation reaction converting diacylglycerol to PA is catalyzed by the diacylglycerol kinase Dgk1 [25,28,29].

This review summarizes our current knowledge about the enzymes involved in PA biosynthesis in the model organism yeast *Saccharomyces cerevisiae*. A special focus is laid upon the regulation and localization of the PA forming enzymes. Similarly, open questions of PA biosynthesis and research needs for a better understanding of the high complexity of lipid metabolism are discussed.

## 2 Two glycerol-3-phosphate acyltransferases initiating PA biosynthesis in yeast

In yeast, biosynthesis of PA is initiated either by acylation of the glycerol backbone of glycerol-3-phosphate or of dihydroxyacetone phosphate (see Fig. 1A). Several details about the enzymes mediating these first reactions of PA biosynthesis were already unveiled at the end of the last century [22,30–33], however, it was only in 2001 that Zheng and Zou identified the ORFs YBL011W and YKR067W as those encoding the major GPATs in yeast, Sct1 (Gat2) and Gpt2 (Gat1) [11]. These two acyltransferases share 37% sequence identity and 54% similarity [11,34]. Most probably, Sct1 and Gpt2 are the only acyltransferases initiating PA biosynthesis in yeast, because a mutant lacking both *SCT1* and *GPT2* is not viable. In contrast to the double deletion mutant, under standard growth conditions both single deletion mutants *sct1*Δ and *gpt2*Δ proliferate like wild type, showing that one acyltransferase can compensate a defect in its counterpart. Nevertheless, detailed analysis of the single deletion mutants unveiled clear differences in the characteristics of Sct1 and Gpt2, and each acyltransferase harbors specific abilities which renders it unique.

### 2.1 Characteristics of the glycerol-3-phosphate acyltransferase Sct1

Although the acyltransferase Sct1 is capable to initiate PA biosynthesis via both the glycerol-3-phosphate pathway and the dihydroxyacetone phosphate pathway (see Fig. 1A), it clearly prefers glycerol-3-phosphate over dihydroxyacetone phosphate as a substrate [11]. Similarly, Sct1 exhibits a distinct preference toward its second substrate, the acyl-CoA species. Among the spectrum of activated fatty acids Sct1 selects preferentially palmitoyl-CoA for incorporation into glycerolipids, which becomes evident upon manipulation of the expression level of *SCT1* [11,26,35]. Whereas mutant cells lacking *SCT1* show a marked reduction in the relative abundance of palmitic acid (C16:0) bound in glycerophospholipids and triacylglycerols [11,26,35], high level expression of *SCT1* dramatically increases the incorporation of this fatty acid species [26]. These changes in the fatty acid pattern of glycerolipids root in a competition between Sct1 and the fatty acid desaturase Ole1 for their shared substrate palmitoyl-CoA [26]. In wild type the incorporation of palmitoyl-CoA into glycerophospholipids and its flux toward fatty acid elongation/desaturation prior to incorporation is balanced, which is important for proper membrane function. However, this balance is lost and shifted either toward a preferential incorporation of palmitoyl-CoA into glycerolipids in cells expressing *SCT1* at high level, or toward elongation/desaturation in *sct1*Δ cells. Noteworthy, the dramatically increased degree of saturation of glycerophospholipids under the former condition strongly compromises cell growth [26,36].

**Fig. 2:**
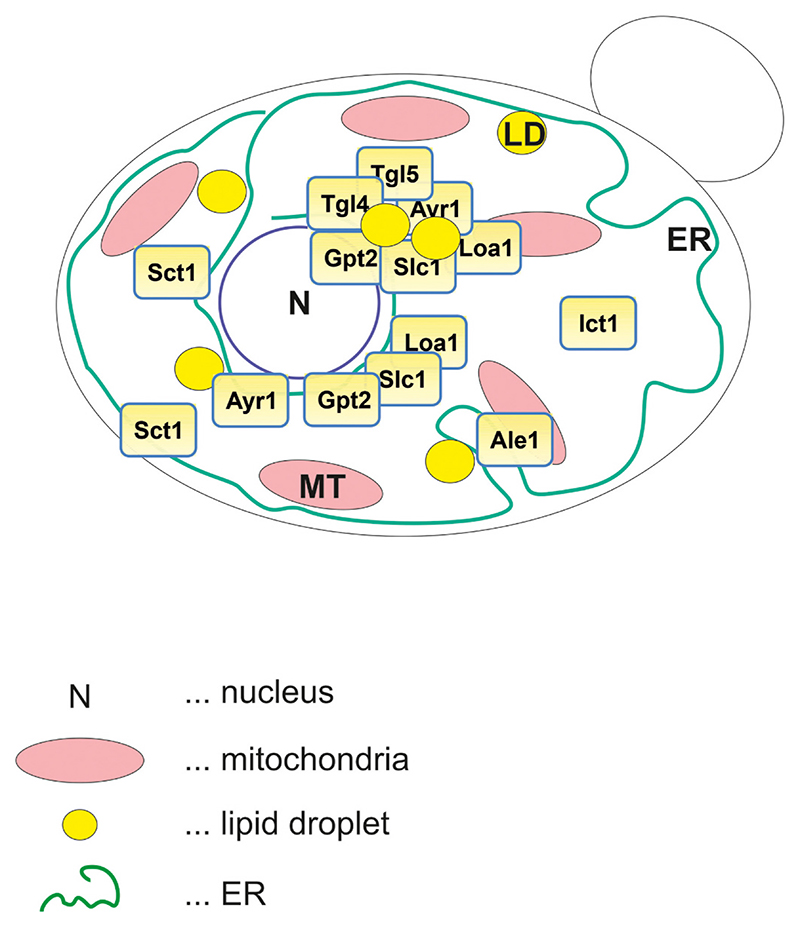
Localization of enzymes contributing to de novo synthesis of phosphatidic acid in yeast. Schema of a yeast cell showing the localization of enzymes contributing to de novo synthesis of phosphatidic acid (PA). Note: for the sake of clarity only relevant structures of the cell are shown, i.e., the endoplasmic reticulum (ER, green line), lipid droplets (LD, yellow spheres), mitochondria (MT, red ellipsoids) and the nucleus (N). For further information see text.

The metabolic hub of lipid synthesis is the ER and therefore, it is not surprising that Sct1 also localizes to this organelle (Fig. 2). More specifically, live-cell imaging analysis of cells expressing GFP (green fluorescent protein) tagged Sct1 revealed that this enzyme resides exclusively in the ER with the main portion located to the perinuclear ER and a minor portion to the peripheral ER [36].

Most interestingly, this subcellular distribution is dynamic and changes under certain conditions. For instance, in the absence of its counterpart GPAT Gpt2 a bigger portion of Sct1 localizes to the peripheral ER than in wild-type cells [36]. Similarly, overexpression of *SCT1* increases the relative abundance of the encoded GPAT at the peripheral ER. These alterations in the subcellular distribution may serve as a means to regulate the contribution of Sct1 to lipid metabolism. Upon overexpression of *SCT1* a bigger portion of the encoded GPAT may localize to the cell periphery to limit the number of Sct1 molecules at the perinuclear ER, which compete with Ole1 and other enzymes for the cellular pool of palmitoyl-CoA. For the same reason Sct1 may be shifted to the cell periphery in cells lacking *GPT2*. The second GPAT Gpt2 uses a broad spectrum of acyl-CoA species as substrate and thus consumes the products of enzymes competing with Sct1 for palmitoyl-CoA. Accordingly, Gpt2 exerts a “pulling function” on these competing reactions and turns the respective enzymes governing these reactions into stronger competitors of Sct1. Lack of *GPT2*, on the other hand, weakens the competing function of these enzymes, and again as a means to keep the balance a higher portion of Sct1 is transferred to the cell periphery. If true, this regulatory mechanism is quite efficient, because the fatty acid pattern of *gpt2*Δ cells is similar to wild type [26] although Sct1 with its pronounced preference for palmitoyl-CoA is the only GPAT contributing to PA biosynthesis in this mutant.

Interestingly, these changes in the intracellular distribution of Sct1 are accompanied by changes at the molecular level. High level expression of *SCT1* markedly decreases the electrophoretic mobility of the encoded GPAT on SDS-gels compared to control, an effect which has been pinpointed to a change in the phosphorylation status of Sct1 [36]. Similarly, the absence of the counterpart GPAT Gpt2 affects the phosphorylation status of Sct1 [36]. But in contrast to *SCT1* overexpression, in *gpt2*Δ cells Sct1 shows increased electrophoretic mobility on SDS-gels compared to wild-type control. The finding that in both, in cells either lacking *GPT2* or overexpressing *SCT1*, a higher portion of Sct1 localizes to the peripheral ER, but the impact on the phosphorylation status of Sct1 differs, indicates that the regulation of Sct1 by posttranslational modification and via the intracellular distribution occurs independently of each other. Changes in the phosphorylation status of Sct1 were similarly observed during investigations performed in the group of A.I. De Kroon [26]. This group reported that in addition to the Sct1-control signal, in cells grown in media supplemented with palmitic acid a marked portion of Sct1 shows a reduced electrophoretic mobility on SDS-gels [26]. Similarly, in some deletion mutants the phosphorylation status of Sct1 is affected. For instance, in a mutant lacking the gene *PSI1/CST26* which encodes an enzyme involved in remodeling of phosphatidylinositol [37], the faster migrating hypo-phosphorylated control form of Sct1 is virtually absent, and only a Sct1-form showing reduced electrophoretic mobility on SDS-gels is observed [26]. Interestingly, in the *psi1*Δ mutant not only the phosphorylation status of Sct1 differs from control, but also the amount of this GPAT is dramatically reduced. This latter finding suggests that phosphorylation and de-phosphorylation at least at one amino acid serves as a means to regulate the protein stability of Sct1.

A prominent role in the regulation of Sct1 may be ascribed to the C-terminus of this enzyme. This portion of the GPAT is rich in the amino acids proline (P), glutamic acid (E), serine (S) and threonine (T), which indicates a PEST region. Since PEST regions are said to be involved in a fast turnover of proteins [38], it is tempting to speculate that some of the serines and threonines of this region are targets of protein kinases, and that changes in the phosphorylation status at these sites influence the stability of the protein. Another striking and unique characteristic of Sct1 which may be involved in the regulation of the GPAT is a polyglutamic acid (E) track formed of 18 consecutive glutamic acid residues at the very C-terminus.

Taken together, the contribution of Sct1 to lipid/cell metabolism is regulated at least at two different levels, namely at the level of localization and at the molecular level through phosphorylation/de-phosphorylation. The presence of some unique structural characteristics located to the C-terminus of Sct1 may indicate that this part of the protein plays a prominent role in the regulation of this GPAT.

### 2.2 Characteristics of the glycerol-3-phosphate acyltransferase Gpt2

**Fig. 3:**
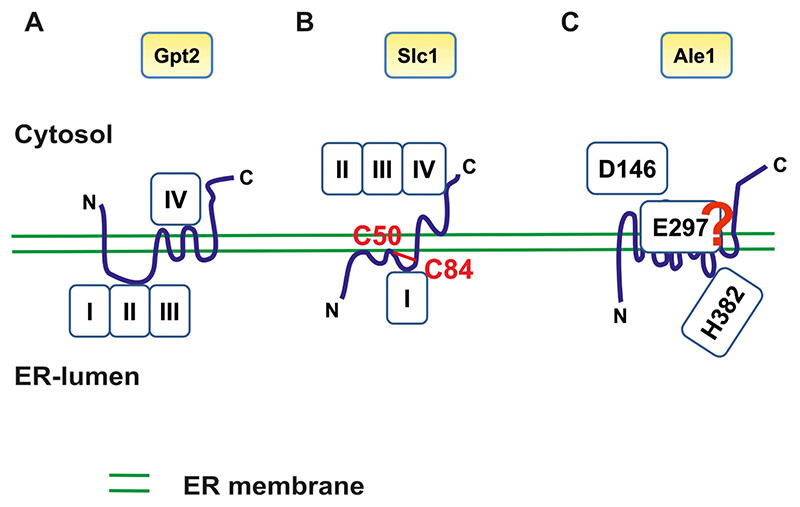
Topology of enzymes contributing to phosphatidic acid biosynthesis in yeast. (A) Model of the topology of the ER associated portion of the glycerol-3-phosphate acyltransferase (GPAT) Gpt2. This model is based on topology information provided by Pagac and colleagues [46]. (B) Model of the major 1-acyl glycerol-3-phosphate acyltransferase (AGPAT) Slc1 which is associated with the ER membrane (according to [47]). In (A) and (B) the sidedness of the conserved motifs (motif I to IV) of the GPAT/AGPAT protein family is shown. Furthermore, in (B) also the localization of two cysteines of Slc1 which form a disulfide bridge is depicted. (C) Topology of the second major AGPAT Ale1 based on the results of topology studies of Pagac and colleagues [47]. In Ale1 the conserved histidine characteristic for the MBOAT protein family is located at position 382 (H382) and oriented toward the ER-lumen. The aspartic acid D146, which has been shown to be important for the catalytic function of Ale1 [48], faces the cytosol, whereas the orientation of the glutamic acid E297, which is also relevant for catalysis, is currently not known. C, C-terminus; N, N-terminus.

Under standard conditions cells lacking *GPT2* grow like wild type, and also high level expression of *GPT2* does not compromise cell growth of the transformants [39]. Likely, the lack of a growth phenotype under the latter condition can be attributed to the broad substrate acceptance of Gpt2. In contrast to Sct1, Gpt2 incorporates a wide range of activated fatty acids into glycerolipids, and also its preference for glycerol-3-phosphate over dihydroxyacetone phosphate is less pronounced than of its counterpart GPAT [11]. In parallel to the broader substrate acceptance, Gpt2 shows also a broader subcellular distribution than Sct1. Like most enzymes contributing to lipid synthesis, Gpt2 resides at the ER (Fig. 2) [27,33,36,39]. However, in addition, a portion of Gpt2 is associated with lipid droplets [27,33,39,40], cell compartments destined for the storage and turnover of non-polar lipids [reviewed in, e. g., [41–45]]. Topology studies of the ER associated portion of Gpt2 revealed that both the N-terminus and the C-terminus of this enzyme are oriented toward the cytosol (Fig. 3A) [46]. Furthermore, with dual topology reporters it was shown that Gpt2 contains 6 transmembrane domains, and that the luminal loop which is formed between transmembrane domain TM1 and TM2 bears the characteristic motifs I, II and III of the GPAT/AGPAT protein family [46]. Strikingly, motif IV which is like motif I involved in catalysis, was found to be oriented toward the cytoplasmic side of the ER and is thus separated from the other motifs by the ER membrane. However, these topology studies were performed with Gpt2 expressed at high level, a condition which leads to hyperphosphorylation of the enzyme [36]. Thus, it is feasible that triggered by the overexpression, a change in the phosphorylation status of the acyltransferase caused a different topology than under physiological conditions. The topology of the Gpt2-portion attached to lipid droplets has not been determined yet.

Most interestingly, when oleic acid serves as the only carbon source the dual localization of Gpt2 to lipid droplets and the ER is lost and the GPAT exclusively localizes to the ER [27]. Under this condition cell viability strictly depends on the enzyme activity of Gpt2 [27,49] and the formation of triacylglycerols is strongly enhanced. In addition, this cultivation condition affects the phosphorylation status of the protein, leading to increased electrophoretic mobility of the GPAT on SDS-gels compared to control [27]. These observations suggest that the contribution of Gpt2 to lipid metabolism is regulated at two levels, namely by phosphorylation and via the intracellular distribution. Proteomics analysis revealed that Gpt2 is modified by phosphorylation at several amino acids, and most of these phosphorylation sites are located to the cytosolic C-terminus of the enzyme ([39,46]; www.yeastgenome.com). Interestingly, three of the phosphorylation sites located to the C-terminus (S664/S668/S671) form a motif which is highly conserved among GPATs from different yeast species [39]. Recently, Kiegerl and colleagues [39] demonstrated that phosphorylation deficiency at this motif increases not only the electrophoretic mobility of the GPAT on SDS-gels, but also increases the enzyme activity of Gpt2, and selectively augments the triacylglycerol content of the mutant compared to control. All these effects linked to phosphorylation deficiency of Gpt2 at the conserved motif strongly resemble the behavior of native Gpt2 in cells cultivated on oleic acid containing medium. However, despite this high resemblance, the phosphorylation deficient Gpt2 mutant form still localizes to lipid droplets. Since phosphorylation deficiency at amino acids different from the conserved motif similarly increases the electrophoretic mobility of Gpt2 on SDS-gels (Kiegerl and Athenstaedt, unpublished results; [50]), the question remains whether phosphorylation/de-phosphorylation is involved in determining the intracellular distribution of this GPAT or not.

To date, the significance of the dual distribution of Gpt2 to lipid droplets and the ER is not well understood. However, the coordination of the enzyme activity of Gpt2 and its distribution between these two organelles seems to be of high importance. Analysis of the lipid droplet proteome revealed that essentially the whole set of enzymes required for the synthesis and degradation of triacylglycerols resides on this organelle. These enzymes comprise the major triacylglycerol lipases Tgl3, Tgl4, Tgl5 cleaving off a fatty acid of triacylglycerols [51–53], the fatty acid activating enzymes Faa1, Faa4 and Fat1, which re-activate fatty acids for re-incorporation into lipids [40,54–57], and the acyltransferases Gpt2, Slc1 and Dga1, which catalyze the consecutive acylation reactions in triacylglycerol synthesis [33,39,58]. Finally, the phosphatidate phosphatase Pah1, which mediates the conversion of PA to diacylglycerol in de novo synthesis of triacylglycerols, has been located to the lipid droplet − ER interface [59–61]. Thus, particularly because of the physical closeness of all these enzymes at the surface of lipid droplets, triacylglycerol synthesis and triacylglycerol degradation need to be strictly coordinated to avoid a futile cycle in which these two processes occur in parallel. Major points of regulation are the initial reactions of triacylglycerol synthesis and degradation which are catalyzed by Gpt2 and triacylglycerol lipases, respectively. Kiegerl and colleagues [39] showed that upon the initiation of triacylglycerol mobilization in wild-type cells Gpt2 gets rapidly phosphorylated to reduce the enzyme’s activity and to adapt the amount of PA formed to the flux toward the glycerophospholipid forming pathways. However, in a mutant expressing the Gpt2-variant which is phosphorylation deficient at the conserved motif S664/S668/S671, in this phase the GPAT activity remains increased. Hence, a higher amount of PA is formed, and the extra portion of PA serves as a substrate for triacylglycerol synthesis. Accordingly, triacylglycerol degradation and triacylglycerol synthesis occur in parallel which results in a futile cycle and thereby counteracts triacylglycerol mobilization [39].

Another consequence of phosphorylation deficiency of Gpt2 at the conserved motif S664/S668/S671 is a strongly increased relative amount of palmitic acid incorporated into triacylglycerols [39]. Interestingly, this is also true for glycerophospholipids although the total amount and the pattern of these membrane lipids are similar to control. Again, these alterations in the fatty acid pattern of glycerolipids are caused by the increased enzyme activity of the phosphorylation deficient Gpt2-variant compared to control, because this hyperactivity turns the GPAT into a serious competitor of the fatty acid desaturase Ole1 for the shared substrate palmitoyl-CoA [39].

Protein-protein interaction represents another means which regulates the contribution of Gpt2 to lipid/cell metabolism. A prominent interaction partner of Gpt2 is seipin (Sei1) [3,62], a protein which is involved and highly relevant in lipid droplet biogenesis [reviewed in, e. g., [4,44,45,63]]. This interaction is of high importance for proper lipid droplet formation, because Sei1 restricts the enzyme activity of Gpt2 [3]. Thus, a dys-balance in the ratio of Sei1 and Gpt2 either through lack of *SEI1* or overexpression of *GPT2* increases the cellular GPAT activity which leads to PA accumulation in the ER, and as a consequence triggers the formation of supersized lipid droplets. Co-overexpression of *GPT2* and *SEI1* or deletion of both genes, on the other hand, markedly reduces the number of supersized lipid droplets in the respective cells [3]. A second prominent interaction partner of Gpt2 is Ldb16 [3], a protein which occurs in complex with Sei1 [60,64]. Whether the interaction between Gpt2 and Ldb16 similarly affects the enzyme activity of the GPAT is not known, because addressing this question would require measurements in the absence of Sei1. However, under these conditions Ldb16 is highly instable and gets rapidly degraded [64].

Taken together, the contribution of Gpt2 to lipid/cell metabolism is regulated at least at three different levels, namely by changing the intracellular distribution (ER - lipid droplets), phosphorylation, and protein-protein interaction. Additional levels of regulation may be unveiled in the future, since in cells challenged by oleic acid the protein abundance of Gpt2 markedly increases [27] which indicates transcriptional regulation.

## 3 Yeast’s 1-acyl glycerol-3-phosphate acyltransferases

1-acyl glycerol-3-phosphate acyltransferases (AGPATs) are also known as lyso-PA acyltransferases (LPAATs). These enzymes mediate the conversion of lyso-PA to PA, which is the ultimate acylation reaction of both, the glycerol-3-phosphate pathway and the dihydroxyacetone phosphate pathway (see Fig. 1A). Also for this reaction enzymes with overlapping function exist, − two major AGPATs, i.e., Slc1 and Ale1, as well as some minor ones. However, the contribution of these minor AGPATs to PA biosynthesis seems to be only relevant under certain conditions and to a limited extent, because a *slc1Δale1*Δ double mutant is not viable [13–15,65].

### 3.1 Characteristics of the major 1-acyl glycerol-3-phosphate acyltransferase Slc1

In yeast, the first enzyme involved in PA biosynthesis identified by gene and function was the AGPAT Slc1 [12]. Originally, Slc1 was picked in a screen for suppressors of yeast strains defective in sphingolipid synthesis. The reason for this hit was a mutation in the *SLC1* gene resulting in the amino acid exchange glutamine to leucine (Q44L) in the encoded AGPAT and thereby affecting the substrate specificity of this enzyme. More precisely, by the Q44L substitution Slc1 formed PA species containing a long chain fatty acid (hexacosanoic acid; C26:0) at the *sn*-2 position. The subsequent use of these PA species for the synthesis of phosphatidylinositol resulted in novel derivatives which compensated the lack of sphingolipids in the mutant used in this screen. This characteristic was also the reason for naming the enzyme Slc1 which stands for *s*phingo*l*ipid *c*ompensation 1 [12]. In contrast to the mutant form, native Slc1 seems to be relevant for the incorporation of short chain acyl-CoAs as well as oleoyl-CoA into glycerolipids, because lipidome analysis detected a significant decrease in the relative abundance of capric acid (C10:0), lauric acid (C12:0), myristic acid (C14:0), and oleic acid (C18:1) in glycerophospholipids of a *slc1*Δ mutant [66]. Oelkers and Pokhrel [35] for their part found that Slc1 preferentially pairs fatty acids of different lengths in PA, meaning that the fatty acid species already linked to the *sn*-1 position of lyso-PA influences the acyl-CoA species selected by Slc1. For instance, Slc1 preferentially links an acyl-CoA species with 18 carbon atoms to C16-lyso-PA, and conversely a C16 acyl-CoA species to C18-lyso-PA. For this reason, information about the fatty acid specificities of Slc1 determined in vitro has to be considered with caution. Concerning its second substrate, the acyl-CoA acceptor, Slc1 exhibits a clear preference for lyso-PA [14]. However, at least in vitro this AGPAT accepts to a limited extent also lyso-phosphatidylserine (lyso-PS) and lyso-phosphatidylinositol (lyso-PI) as substrates [14].

Slc1 belongs to the GPAT/AGPAT protein family and is like Gpt2 dually localized to lipid droplets and the ER (Fig. 2) [33]. The topology of the ER associated portion of Slc1 has been partially determined by Pagac and colleagues [47]. This group reported that Slc1 is anchored in the ER membrane by one transmembrane domain. In addition, this AGPAT contains three hydrophobic stretches which are dipping into the membrane bilayer (Fig. 3B). The C-terminus of this AGPAT, which bears the characteristic motifs II, III and IV of the GPAT/AGPAT protein family, is oriented toward the cytosolic side of the ER membrane, whereas motif I is oriented toward the ER lumen. Like for the GPAT Gpt2, separation of the motifs by the ER membrane raises the question as to the catalytic mechanism used by the AGPAT. Furthermore, Pagac and colleagues [47] demonstrated that Slc1 contains one disulfide-bridge, which is formed between the cysteines at position C50 and C84. Interestingly, the latter cysteine (C84) is located close to the catalytically important histidine H82 of motif I. Cysteine C50, on the other hand, is part of the predicted membrane dipping domain TM2. The formation of this disulfide-bridge is irrelevant for the enzymatic function of the protein, since the enzyme is still active upon replacement of these two cysteines (as well as three others) by alanines. However, the formation of this disulfide-bridge may be involved in fine-tuning the activity of Slc1 or in regulating the dual distribution of the protein between lipid droplets and the ER.

A common means to regulate the activity of an enzyme is via a change of its phosphorylation status. This kind of regulation may also be true for Slc1, because this AGPAT has been reported as phospho-protein [67]. Furthermore, in high throughput experiments 7 amino acids of Slc1 have been found to be modified by ubiquitin (www.yeastgenome.org). Since ubiquitin marks a protein for degradation, possibly the contribution of Slc1 to lipid metabolism is regulated via its protein abundance.

### 3.2 Characteristics of the major 1-acyl glycerol-3-phosphate acyltransferase Ale1

Enzyme activity measurements performed with isolated organelles of a *slc1*Δ mutant and control revealed not only that Slc1 is dually localized to lipid droplets and the ER, but also that the latter organelle harbors at least one additional AGPAT [33]. Ten years later, in 2007, several groups reported independently the identification of a second prominent AGPAT, Ale1, which is localized to the ER [13–17]. Ale1 governs not only the acylation of lyso-PA but also of other lyso-phospholipids, in particular lyso-phosphatidylethanolamine (lyso-PE) and lyso-phosphatidylcholine (lyso-PC). These latter substrate preferences were also the reason for the different names originally chosen for this enzyme, which were beside Ale1 [13] Lpt1 [15,17] and Lca1 [16]. Whereas Ale1 stands for *a*cyltransferase for *l*yso-P*E* 1, Lpt1 and Lca1 are different abbreviations for lyso-PC acyltransferase 1. Last but not least, Ale1 is also known as Slc4 [14], a name which was chosen following the abbreviation of the major AGPAT Slc1 [12]. Concerning the preferences toward the second substrate, the acyl-CoA species, Ale1 exhibits a rather low selectivity and accepts activated fatty acids of different lengths and degree of saturation [16].

The broad substrate spectrum of Ale1, particularly toward the acyl-CoA acceptor, may be a consequence of its intracellular localization. Whereas the GPATs Sct1 and Gpt2 localize and produce lyso-PA at the perinuclear ER [36], Ale1 also localizes to the ER but is enriched at contact sites formed between this organelle and mitochondria (MAMs) (Fig. 2) [13]. Accordingly, Ale1 localizes away from the center of lyso-PA production, which may retard its access to this substrate and thereby may have contributed to the development of the broad substrate acceptance of the acyltransferase. In line with a reduced access, Ståhl and colleagues [68] observed that in cell free extracts lyso-PA formed from glycerol-3-phosphate was not efficiently converted to PA by Ale1.

Topology studies revealed that Ale1 is tightly associated with the ER membrane through 13 transmembrane domains (Fig. 3C) [47]. Because of the uneven number of transmembrane domains, the N-terminus and the C-terminus are oriented to the opposite side of the ER membrane, namely the ER lumen and the cytosol, respectively. Consistent with its ER localization, Ale1 bears at its C-terminus the characteristic KKXX retention signal of ER proteins. Searches for other characteristic motifs revealed that Ale1 belongs to the rather diverse group of membrane bound O-acyltransferases (MBOAT protein family). This group is characterized solely by the presence of one conserved histidine. In Ale1 the respective histidine is located at position 382 (H382) which is found in a luminal ER loop connecting the transmembrane domains TM10 and TM11. Currently, it is not clear whether this conserved histidine indeed participates in catalysis or not. Studies of Renauer and colleagues [48], however, identified two amino acid residues of Ale1 which are important for the catalytic activity of the acyltransferase. These two amino acids are an aspartic acid at position 146 (D146) and a glutamic acid at position 297 (E297). The aspartic acid D146 is predicted to be oriented toward the cytoplasmic side of the ER, whereas for E297 different orientations are predicted depending on the algorithm used (Fig. 3C) [48]. Since D146, E297 and the conserved H382 with its questionable role in catalysis are oriented toward different sides of the ER membrane, it is challenging to conceive a catalytic mechanism for the reaction mediated by Ale1 which involves all three amino acids.

To date, the question as to the regulation of Ale1 has not been studied in detail. However, global analysis revealed that Ale1 is a phosphoprotein which is modified by phosphorylation at several sites (www.yeastgenome.org). One challenging task of the future will be to confirm the correctness of this information and to determine the physiological role of these post-translational modifications.

### 3.3 Enzymes exhibiting minor 1-acyl glycerol-3-phosphate acyltransferase activity

Slc1 and Ale1 are the major AGPATs of yeast, since deletion of both *SLC1* and *ALE1* causes lethality [13–15,65]. However, nevertheless several other enzymes exhibiting AGPAT activity have been described. One of these is Loa1 which is also known as Vps66 [21]. This enzyme is a member of the GPAT/AGPAT protein family, but contains only motif I and motif III out of the four characteristic motifs of this group. Most interestingly, in motif I (HX_4_D motif) which is involved in catalysis the conserved histidine is replaced by a cysteine. The official name Loa1 stands for *l*yso-PA:*o*leoyl-CoA *a*cyltransferase 1 and already points to the enzyme’s strong preference for the substrates lyso-PA and oleoyl-CoA [21]. Indeed, Loa1 accepts exclusively lyso-PA as a substrate but no other lyso-phospholipids or glycerol-3-phosphate, monoacylglycerol and diacylglycerol. The pronounced substrate specificity toward oleoyl-CoA, on the other hand, might be the reason for the growth defect of *loa1*Δ cells on media containing oleic acid as a carbon source [49].

Localization studies revealed that Loa1 is dually localized to lipid droplets and the ER (Fig. 2) [21]. Presumably, the association of Loa1 with the surface of lipid droplets is mediated by a hydrophobic stretch present in this AGPAT. The 1 to 2 predicted transmembrane domains (depending on the algorithm used [21]), on the other hand, may be relevant for the enzyme’s association with the ER membrane. Most interestingly, the distribution of this AGPAT between lipid droplets and the ER depends on the growth phase [21,40]. During exponential growth Loa1 localizes to the ER, whereas in stationary phase this protein additionally associates with lipid droplets [21]. Furthermore, it was shown that the absence/presence of Loa1 affects the cell morphology. Compared to wild type, *loa1*Δ cells are bigger in size and contain a higher number of small lipid droplets [21]. But despite the higher number of lipid droplets, during exponential growth as well as in stationary phase the cellular triacylglycerol level of *loa1*Δ is reduced. This finding points to a contribution of Loa1 to triacylglycerol synthesis − maybe particularly when cells are challenged by oleic acid, since this condition compromises the growth of cells lacking *LOA1* [49].

To date, no phosphorylation sites have been reported for Loa1 ([67]; www.yeastgenome.org). However, this enzyme is modified by ubiquitin (www.yeastgenome.org), which suggests that the contribution of Loa1 to cell metabolism may be regulated via this protein modification.

Another minor AGPAT expressed in yeast is Ict1 [20]. This enzyme belongs to the GPAT/AGPAT protein family but contains only motif I (HX_4_D motif) out of the four characteristic motifs of this protein family. Interestingly, in contrast to most GPATs/AGPATs, in Ict1 the HX_4_D motif is found in the C-terminal portion of the protein instead of in the N-terminal one. This finding may indicate that Ict1 is a remnant of a former full size GPAT/AGPAT. Ict1 seems to play a specific role in PA biosynthesis in cells challenged with the organic solvent iso-octanol [20]. However, since exposure of *ict1*Δ cells to iso-octanol does not result in lyso-PA accumulation, obviously also other AGPATs markedly contribute to PA biosynthesis under this condition. In vitro, Ict1 selectively acylates lyso-PA but no other lyso-phospholipids [20]. In addition, it was shown that oleoyl-CoA is the preferred acyl-CoA moiety used by this AGPAT. However, Ict1 also links palmitoyl-CoA and to a lesser extent stearoyl-CoA to the glycerol backbone of lyso-PA.

Like Loa1, Ict1 lacks any phosphorylation sites, but similarly no ubiquitination sites have been reported for this AGPAT ([67]; www.yeastgenome.org). Furthermore, computational searches for topological relevant domains revealed that Ict1 lacks transmembrane domains, which indicates a cytosolic localization (Fig. 2) [20]. However, the precise intracellular localization of this AGPAT has not been determined yet.

Among the minor AGPATs contributing to PA biosynthesis are also the lipid droplet associated triacylglycerol lipases Tgl4 and Tgl5 (Fig. 2) [18,19,52,69]. In silico analysis of the sequence of Tgl5 revealed that this enzyme bears not only the conserved lipase motif GXSXG, but also a HX_4_D motif which is a characteristic of numerous acyltransferases [18]. Indeed, by testing the purified enzyme for acyltransferase activity Rajakumari and Daum [18] measured such an activity when lyso-PA served as a substrate. These authors also tested the substrate preference toward the acyl-CoA species and showed that Tgl5 prefers oleoyl-CoA over palmitoyl-CoA. Furthermore, high throughput experiments revealed that Tgl5 is targeted by protein kinases (www.yeastgenome.com). Whether a change in the phosphorylation status is involved in switching the function of this enzyme from a triacylglycerol lipase to an AGPAT remains to be tested.

Tgl4 shares considerable homology with Tgl5 and similarly exhibits AGPAT activity [19]. But surprisingly, this triacylglycerol lipase lacks the characteristic acyltransferase motif HX4D, and the amino acids, which are relevant for the conversion of lyso-PA to PA, are currently not known. Like Tgl5, Tgl4 exhibits a strong preference for the substrate lyso-PA, whereas other lyso-phospholipids are hardly metabolized by this enzyme [19]. The preferred acyl-CoA species used by Tgl4 for the acylation reaction is oleoyl-CoA. Furthermore, proteomics analysis revealed that Tgl4 is targeted by protein kinases at several amino acid residues ([67]; www.yeastgenome.com). To date, two of these phosphorylation sites have been shown to be important for regulating the lipase function of the enzyme, but not the AGPAT function [19,70]. Whether phosphorylation/de-phosphorylation at one of the other phosphorylation sites controls the AGPAT function of Tgl4 remains to be investigated.

## 4 1-Acyl dihydroxyacetone phosphate reductases and characteristics of the 1-acyl dihydroxyacetone phosphate reductase Ayr1

In contrast to the acyltransferase reactions of PA biosynthesis which are common to both, the glycerol-3-phosphate pathway and the dihydroxyacetone phosphate pathway, the 1-acyl dihydroxyacetone phosphate reductase reaction is specific for the latter pathway (see Fig. 1). To date, the only enzyme mediating this reaction which has been identified at the molecular level, is the 1-acyl dihydroxyacetone phosphate reductase Ayr1 [23]. However, since minor 1-acyl dihydroxyacetone phosphate reductase activity is still measured in a strain devoid of *AYR1*, the encoded enzyme has at least one counterpart which remains to be identified.

Localization studies revealed that Ayr1 is dually localized to lipid droplets and the ER (Fig. 2) [23]. The association with the former organelle may involve two hydrophobic stretches which are located to the middle and the C-terminus of Ayr1, respectively [23]. To catalyze the reduction of 1-acyl dihydroxyacetone phosphate to lyso-PA, Ayr1 requires as reducing agent NADPH. The binding site for this compound is located to the N-terminus between amino acids 13 to 37. Interestingly, this region bears similarly a GXSXG motif which is characteristic for lipases. Indeed, the serine of this motif (S18) has been shown to be relevant for a minor contribution of Ayr1 to triacylglycerol degradation [71].

The question as to the regulation of Ayr1 has not been studied yet. However, the contribution of this enzyme to PA biosynthesis may be regulated by phosphorylation, because high throughput experiments identified Ayr1 as target of a protein kinase. The detected phosphorylation site is a serine, which is located to the C-terminus of the reductase (www.yeastgenome.com). In addition, several lysines of Ayr1 have been reported to be modified by ubiquitin. Since the protein amount of Ayr1 increases during growth [72], this post-translational modification may be involved in regulating the abundance of this enzyme.

A short summary of characteristics of the enzymes involved in PA biosynthesis is provided in Table 1.

## 5 Comments and open questions

Biosynthesis of PA is prerequisite for the de novo formation of all glycerolipids, i.e., glycerophospholipids and triacylglycerols. As outlined above, to date all major as well as several minor enzymes contributing to PA biosynthesis have been identified at the molecular level. However, our knowledge about the regulation of these enzymes, which is vital for understanding the high complexity of the lipid metabolic network, is still very limited. Thus, one focus of future studies should be laid upon elucidating regulatory aspects of PA biosynthesis. High throughput experiments investigating post-translational modifications revealed that most enzymes involved in PA biosynthesis are modified by phosphorylation. A change in the phosphorylation status of an enzyme regulates its contribution to metabolism for instance by affecting the enzyme activity, protein stability, and/or localization. Presently, only the significance of one single conserved phosphorylation motif of the major GPAT Gpt2 has been studied in detail. This study showed that phosphorylation/de-phosphorylation at this motif affects the enzyme’s activity [39] (see Section 2.2.). However, beside this motif Gpt2 contains several other phosphorylation sites, raising the question as to the significance of phosphorylation/de-phosphorylation at these sites. One study already showed that in cells shifted from standard medium to oleic acid containing medium the phosphorylation status of Gpt2 changes [27]. Interestingly, at the same time the GPAT loses its dual distribution between lipid droplets and the ER in favor of the latter organelle. These observations suggest that a change in the phosphorylation status of Gpt2 at certain phosphorylation sites directs the sub-cellular distribution of this enzyme.

Interestingly, not only Gpt2 is dually localized to lipid droplets and the ER, but also the AGPATs Slc1 and Loa1, as well as the 1-acyl dihydroxyacetone phosphate reductase Ayr1 [21,23,33]. Thus, yeast contains one entire set of PA synthesizing enzymes at two different locations. By elucidating the significance of this dual localization we will not only immensely contribute to our understanding of PA biosynthesis, but also unravel the role of lipid droplets in this process. For instance, it may turn out that this set of PA synthesizing enzymes uses lipid droplets just as a parking lot and is shifted between lipid droplets and the ER according to cellular needs.

Another interesting topic which should be addressed in the future concerns the association of PA synthesizing enzymes with lipid droplets. For the ER associated portion of Gpt2 it has been shown that this GPAT contains 6 transmembrane domains [46]. Similarly, both AGPATs, Slc1 and Loa1, are anchored to the ER by at least one transmembrane domain [21,47]. However, since these structures are incompatible with an association with the monolayer membrane surrounding a lipid droplet, the question remains as to the attachment of these enzymes to the surface of the droplet. Similarly, our current knowledge about structural features of other PA synthesizing enzymes needs to be complemented. For instance, a striking characteristic of the second GPAT Sct1 is a polyglutamic acid (E) track located to the C-terminus (see Section 2.1.). Because overexpression of both, native Sct1 and a Sct1 mutant form lacking the poly-glutamic acid track, rescues a strain defective in a choline transporter from lethality [38], this structural feature does not seem to be directly involved in regulating the enzyme activity of this GPAT. However, the question remains whether the poly-glutamic acid track is involved in regulating the localization, protein stability, and/or protein-protein interactions of Sct1.

Last but not least, also minor enzymes contributing to PA biosynthesis should not be lost out of focus, because the knowledge about these enzymes, their features and regulation will be similarly important to complement our current picture of PA biosynthesis. Collectively, by shedding light on the entire set of enzymes involved in PA biosynthesis, their regulation, their structural features, but also their interplay, we will be able to contribute important information toward a better understanding of PA biosynthesis, a centerpiece of the overall complex network of lipid metabolism.

## Figures and Tables

**Table 1:** Characteristics of enzymes involved in PA biosynthesis.

Protein (synonyms)	ORF/Gene	Molecular mass/protein half-life time	Substrate preferences	Number of (predicted) transmembrane domains	Other characteristics	Post translational modifications	Localization	Protein family
Sct1 (Gat2)	YBL011W/ *SCT1*	85.7 kDa ~7.6 h	Palmitoyl-CoA, G-3-P>>DHAP	3–6	HX_5_D motif, polyglutamic acid track, PEST − region (C-terminus)	Phosphorylation	ER	GPAT/AGPAT
Gpt2 (Gat1)	YKR067W/ *GPT2*	83.7 kDa ~6.8 h	G-3-P>DHAP	6	HX_5_D motif, serine rich C-terminus	Phosphorylation	ER, LD	GPAT/AGPAT
Slc1	YDL052C/ *SLC1*	33.9 kDa ~8.3 h	Pairing acyl-chains of different lengths lyso-PA>>lyso-PS, lyso-PI	1		Phosphorylation (predicted), ubiquitination (predicted)	ER, LD	GPAT/AGPAT
Ale1 (Lpt1, Slc4, Lca1)	YOR175C/ *ALE1*	72.3 kDa ~20 h	Lyso-PE, lyso-PC>>lyso-PA	13		Phosphorylation (predicted)	ER (MAM)	MBOAT
Ict1	YLR099C/ *ICT1*	45.1 kDa ~8.3 h	Oleoyl-CoA>palmitoyl-CoA> stearoyl-CoA lyso-PA	None	HX_4_D motif	–	Cytosolic (predicted)	GPAT/AGPAT
Loa1 (Vps66)	YPR139C/ *LOA1*	33.8 kDa ~13.1 h	Oleoyl-CoA lyso-PA	1-2	motifs I + III, motif I (CX_4_D)	Ubiquitination (predicted)	ER, LD	GPAT/AGPAT
Tgl4	YKR089C/ *TLG4*	102.7 kDa ~4.2 h	Oleoyl-CoA lyso-PA	None		Phosphorylation	LD	Lipase protein family
Tgl5	YOR081C/ *TGL5*	84.7 kDa ~2.1 h	Oleoyl-CoA>palmitoyl-CoA lyso-PA	None	HX_4_D motif	Phosphorylation (predicted)	ER, LD	Lipase protein family
Ayr1	YIL124W/ *AYR1*	32.8 kDa ~8.0 h		None	NADPH binding site, GXSXG motif	Phosphorylation (predicted), ubiquitination (predicted)	ER, LD	Reductase protein family

DHAP, dihydroxyacetone phosphate; ER, endoplasmic reticulum; G-3-P, glycerol-3-phosphate; LD, lipid droplet; lyso-PC, lyso-phosphatidylcholine; lyso-PE, lyso-phosphatidylethanolamine; lyso-PI, lyso-phosphatidylinositol; lyso-PS, lyso-phosphatidylserine; MAM, ER associated mitochondrial fraction.

## Abbreviations

AGPAT: 1-acyl glycerol-3-phosphate acyltransferase
ER: endoplasmic reticulum
GPAT: glycerol-3-phosphate acyltransferase
lyso-PA: lyso-phosphatidic acid
lyso-PC: lyso-phosphatidylcholine
lyso-PE: lyso-phosphatidylethanolamine
lyso-PI: lyso-phosphatidylinositol
lyso-PS: lyso-phosphatidylserine
MBOAT: membrane bound O-acyltransferase
PA: phosphatidic acid
TM: transmembrane domain
